# Chronic post‐operative opioid use after open cardiac surgery: A Danish population‐based cohort study

**DOI:** 10.1111/aas.13688

**Published:** 2020-09-09

**Authors:** Kasper Bonnesen, Lone Nikolajsen, Henrik Bøggild, Per Hostrup Nielsen, Carl‐Johan Jacobsen, Dorthe Viemose Nielsen

**Affiliations:** ^1^ Department of Clinical Epidemiology Aarhus University Hospital Aarhus Denmark; ^2^ Department of Anesthesiology and Intensive Care Aarhus University Hospital Aarhus Denmark; ^3^ Public Health and Epidemiology Department of Health Science and Technology Aalborg University Aalborg Denmark; ^4^ Unit of Clinical Biostatistics Aalborg University Hospital Aalborg Denmark; ^5^ Department of Cardiothoracic Surgery Aarhus University Hospital Aalborg Denmark

## Abstract

**Background:**

Knowledge of chronic opioid use after cardiac surgery is sparse. We therefore aimed to describe the proportion of new chronic post‐operative opioid use after open cardiac surgery.

**Methods:**

We used prospectively registered data from a national prescription registry and a clinical registry of 29 815 first‐time cardiac surgeries from three Danish university hospitals. Data collection spanned from 2003 to 2016. The main outcome was chronic post‐operative opioid use, defined as at least one opioid dispensing in the fourth post‐operative quarter. Data were assessed for patient‐level predictors of chronic post‐operative opioid use, including pre‐operative opioid use, opioid use at discharge, comorbidities, and procedural related variables.

**Results:**

The overall proportion of post‐operative opioid use was 10.6% (95% CI: 10.2‐10.9). The proportion of new chronic post‐operative opioid use was 5.7% (95% CI: 5.5‐6.0) among pre‐operative opioid naïve patients. The corresponding proportions among patients, who pre‐operatively used low or high dose opioid (1‐500 mg or > 500 mg cumulative morphine equivalent opioid), were 68.3% (95% CI: 66.1‐70.4) and 76.3% (95% CI: 74.0‐78.5) respectively. Risk factors associated with new chronic post‐operative opioid use included: female gender, underweight and obesity, pre‐operative comorbidities, acute surgery, ICU‐time > 1 day, and post‐operative complications. Strongest predictor of chronic post‐operative opioid use was post‐discharge use of opioid within one month after surgery (odds ratio 3.3, 95% CI: 2.8‐4.0).

**Conclusion:**

New chronic post‐operative opioid use after open cardiac surgery is common. Focus on post‐discharge opioid use may help clinicians to reduce rates of new chronic opioid users.

## 1 INTRODUCTION

Opioid use is a serious healthcare concern globally as well as in Denmark.^1^, ^2^, ^3^ Denmark has one of the highest rates of opioid consumption in the world with 3%‐5% of all Danes using opioid regularly.^4^ This is concerning as chronic opioid use is associated with increased risk of opioid‐related morbidity and mortality.^5^, ^6^, ^7^ As one common indication for opioid prescriptions, special attention should be devoted toward opioid prescribing following surgical procedures.^8^

In Denmark, approximately 4000 cardiac surgeries involving sternotomy are performed annually.^9^ The use of intraoperative opioid treatment following cardiac surgery is necessary to alleviate pain caused by artery harvesting, rib retraction, sternotomy, and drain tubes.^10^ Effective intraoperative pain management may reduce post‐operative complications such as myocardial infarction, cardiac arrhythmias, hypercoagulability, and pulmonary complications.^10^, ^11^ While opioids may be necessary to control pain in the intraoperative period, studies suggest that the harms of opioid use after hospital discharge may outweigh the benefits, as post‐operative opioid use has been associated with chronic opioid use, misuse, opioid use disorder, overdose, and cognitive dysfunction.^12^, ^13^, ^14^, ^15^ In fact, several studies show that 3‐10% of patients who did not take opioids before surgery develop a chronic opioid use after surgery.^16^, ^17^, ^18^

Several risk factors for chronic post‐operative opioid use have been identified including male gender, older age, medical and psychiatric comorbidities, concomitant benzodiazepine use, and type and extent of surgery, among others.^16^, ^17^, ^19^, ^20^ Although previous studies have examined the associations between various types of surgeries and long‐term opioid use, the knowledge of chronic post‐operative opioid use after cardiac surgery is sparse.

We aimed to examine patterns of opioid use within the first year after open cardiac surgery and estimate the risk of chronic post‐operative opioid use according to pre‐, intra‐, and post‐operative characteristics.

## 2 MATERIALS AND METHODS

### 2.1 Study design and setting

This population‐based cohort study used prospectively collected data from medical and administrative registries in Western Denmark. Western Denmark has a population of approximately 3.3 million people corresponding to 55% of the total Danish population.^21^ Danish citizens are assigned a unique 10‐digit Civil Personal Register number allowing linkage of Danish registries on an individual level.^22^

The study was approved by the Danish Data Protection Agency (Case no.: 1‐16‐02‐42‐18) and the Danish Patient Safety Authority (Case no. 3‐3013‐2491/1). Approval by the local ethics committee and collection of informed consent are not required for register‐based studies in Denmark.

### 2.2 Data sources

Patients were identified from the Western Denmark Heart Registry (WDHR)—a mandatory internet‐based clinical registry covering all adult patients undergoing cardiac surgery in Western Denmark. Detailed patient, surgery, anesthesia, and intensive care data are prospectively collected on consecutive patients.^21^, ^23^ Data on opioid dispensing were retrieved from the National Prescription Registry (NPR)—a registry containing detailed nationwide information about patient, dispensing, prescriber, and pharmacy information on redeemed prescriptions since 1995.^24^ Data in both WDHR and NPR are considered both complete and valid.^21^, ^23^, ^25^ Baseline characteristics were identified from the WDHR using EuroSCORE I^15^ and 2013 EuroSCORE II.^16^ The specific definitions of the Euroscore I and II can be seen elsewhere.^15^ ^;^ ^16^

The Danish Health Service provides universal tax‐supported healthcare, guaranteeing all Danish residents free access to general practitioners and hospitals. In Denmark, general practitioners issue most prescriptions. Patient co‐payment is required for prescription drugs. Any reimbursable medicines are covered by a tax‐financed drug reimbursement scheme.^26^

### 2.3 Study population

**Figure 1:**
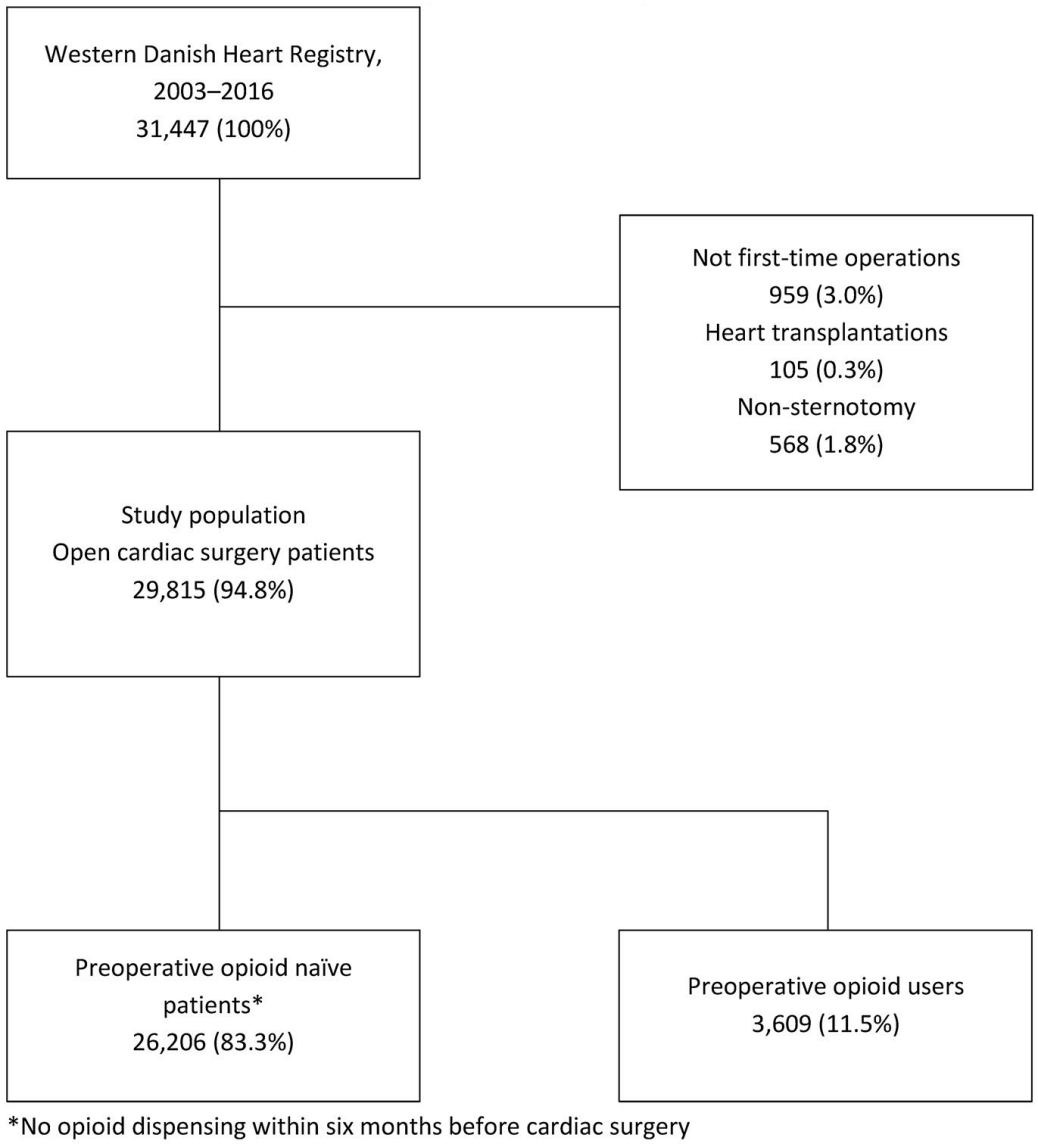
Flowchart of cohort inclusion, western Denmark, 2003–2016. *No opioid dispensing within six months before cardiac surgery

The study included all first‐time cardiac surgical procedures conducted in the three university hospitals in Western Denmark from January 1, 2003 to December 31, 2016. Availability of data in the Danish Prescription Database defined the chosen time‐period. Prior to study‐time restriction, patients with missing data on time of surgery (n = 7) and patients with an invalid Central Personal Registration number were excluded (n = 223). After study‐time restriction, all non‐first‐time procedures, or if the surgical procedure did not involve sternotomy or was a heart transplantation were excluded (Figure 1).

All patients completed the 1‐year follow‐up. Missing data on pre‐, intra‐, and post‐operative characteristics ranged from 2.5% to 8.4%.

### 2.4 Outcome

Opioid dispensings were identified from 6 months before surgery to 12 months after surgery. We defined the primary outcome “chronic post‐operative opioid use” a priori before data extraction. We divided opioid use into four 3‐month periods (quarters) after surgery and defined opioid use as at least one opioid dispensing in one of these quarters. We defined chronic post‐operative opioid use as at least one opioid dispensing in the fourth post‐operative quarter. We defined late initiators as individuals with a first opioid dispensing later than three months after surgery.

### 2.5 Covariates

Included covariates were defined a priori before data extraction. Pre‐operative opioid use was included as a possible risk factor for chronic post‐operative opioid use. Pre‐operative opioid use was defined as at least one opioid dispensing within 6 months before cardiac surgery. We further divided pre‐operative opioid use into low (≤500 mg) or high dose (>500 mg) based on cumulative morphine equivalent opioid use within 6 months before cardiac surgery. Patients’ descriptive and clinical characteristics were collected at the time of cardiac surgery. Pre‐operative demographic and clinical characteristics included gender, age group (<60, 60‐70, and > 70), BMI (<18.5, 18.5‐24.9, 25.0‐29.9, and > 30.0), chronic obstructive pulmonary disease, peripheral artery disease, chronic kidney failure, diabetes, left ventricle ejection fraction (≥50, 31‐50, and ≤ 30), and pulmonary hypertension. Intraoperative characteristics included acute surgery, surgery type (coronary artery bypass grafting without valve surgery and combinations and other), epidural anesthesia, extra corporal circulation, and intensive care unit (ICU) time more than one day. Post‐operative characteristics included myocardial infarction, renal replacement therapy, reoperation, sternal wound infection, and stroke. We defined post‐discharge opioid use as cumulative morphine equivalent opioid (OME) use within one month after cardiac surgery and divided this into 0, 1‐500 mg, and > 500 mg.

### 2.6 Statistical analyses

Proportions and numbers are used to describe the study population stratified by opioid naïve patients and opioid users before surgery. Categorical data are presented as numbers and percentages (%) with an estimated 95% confidence interval (CI). Comparisons of categorical data were made using a chi‐squared test and results with a *P*‐value < .05 were considered significant. Continuous data are presented as means with a 95% CI for normally distributed data and as medians with interquartile ranges for skewed data. Patients had to be alive on the last day of each of the four post‐operative quarters to be included in the calculation for that particular period. Logistic regression was applied to estimate odds ratios (OR) and 95% CI of chronic post‐operative opioid use according to pre‐, intra‐, and post‐operative patient characteristics. ORs were stratified according to pre‐operative opioid use in order to minimize any risk of effect modification. Crude estimates with 95% CIs were reported. All statistical analyses were conducted in SAS version 9.4.

## 3 RESULTS

### 3.1 Cohort characteristics

Among 29,815 patients who had open cardiac surgery between 2003 and 2016 in Western Denmark, 26,202 (87.9%) were pre‐operative opioid naïve and 3609 (12.1%) were pre‐operative opioid users (Table 1). Pre‐operative opioid users were more likely to be female, to be obese, or to have a diagnosis of chronic obstructive pulmonary disease, peripheral artery disease, diabetes, or pulmonary hypertension (all *P* < .01). Less pre‐operative opioid users were alive and the end of the fourth post‐operative quarter (90% compared to 94% of pre‐operative opioid naïve patients). Pre‐operative opioid users were more likely to have acute surgery, to have an ICU‐time > 1 day, or to be under extracorporeal circulation during the operation (all *P* < .01). Pre‐operative opioid users had a higher frequency of post‐operative myocardial infarction (*P* = .04), renal replacement therapy (0 < 0.01), and sternal wound infection (*P* < .01).

**Table 1:** Characteristics of patients subjected to open cardiac surgery, stratified by pre‐operative opioid status, Western Denmark, 2003‐2016

	All patients, n (%)	Opioid naïve^a^, n (%)	Opioid user, n (%)
Total	29 815 (100)	26 206 (100)	3609 (100)
1a. Baseline characteristics			
Gender			
Female	7753 (26.0)	6486 (24.8)	1267 (35.1)
Missing	928 (3.1)	782 (3.0)	146 (4.1)
Age			
<60 years	6824 (22.9)	6044 (23.1)	780 (21.6)
60−70 years	9228 (31.0)	8197 (31.3)	1031 (28.6)
>70 years	12 971 (43.5)	11 299 (43.1)	1672 (46.3)
Missing	792 (2.7)	666 (2.5)	126 (3.5)
BMI			
<18.5	14.10 (4.7)	1219 (4.7)	191 (5.3)
18.5‐24.9	8582 (28.8)	7620 (29.1)	962 (26.7)
25.0‐29.9	11 523 (38.7)	10 255 (39.1)	1268 (35.1)
>30.0	5993 (20.2)	5109 (19.5)	884 (24.5)
Missing	2307 (7.7)	2003 (7.6)	304 (8.4)
Euroscore			
Median (IQR)	5 (3‐8)	5 (3‐8)	5 (4‐9)
Missing	928 (3.1)	782 (3.0)	146 (4.0)
Chronic obstructive pulmonary disease			
Yes	3,185 (10.7)	2,630 (10.0)	555 (15.4)
Missing	1004 (3.4)	844 (3.2)	160 (4.4)
Peripheral artery disease			
Yes	2807 (9.4)	2309 (8.8)	498 (13.8)
Missing	997 (3.3)	840 (3.2)	157 (4.4)
Chronic kidney failure^b^			
Yes	748 (2.5)	610 (2.3)	138 (3.8)
Missing	1052 (3.5)	889 (3.4)	163 (4.5)
Diabetes^c^			
Yes	4223 (14.2)	3583 (13.7)	640 (17.7)
Missing	1478 (5.0)	1269 (4.8)	209 (5.8)
Left ventricle ejection fraction			
≥50	17 364 (58.2)	15 277 (58.3)	2087 (57.8)
31‐50	9467 (31.8)	8342 (31.8)	1125 (31.2)
≤30	1866 (6.3)	1,640 (6.3)	226 (6.3)
Missing	1118 (3.8)	947 (3.6)	171 (4.7)
Pulmonary hypertension			
Yes	2357 (7.9)	2021 (7.7)	336 (9.3)
Missing	1461 (4.9)	1245 (4.8)	216 (6.0)
Status fourth quarter			
Alive	27 894 (93.6)	24 638 (94.0)	3256 (90.2)
1b. Intraoperative characteristics			
Acute surgery			
Yes	2369 (8.0)	2060 (7.9)	309 (8.6)
Missing	931 (3.1)	784 (3.0)	147 (4.1)
Surgery type			
CABG, without valve surgery	14 869 (49.9)	13 225 (50.5)	1644 (45.6)
Combinations and other	14 124 (47.4)	12 291 (46.9)	1833 (50.5)
Missing	822 (2.8)	690 (2.6)	132 (3.7)
Epidural^d^			
Yes	3588 (36.3)	3199 (36.7)	389 (33.5)
Missing	105 (1.1)	90 (1.0)	15 (1.3)
Extra corporeal circulation			
Off pump	3551 (11.9)	3048 (11.6)	503 (13.9)
Missing	1463 (4.9)	1269 (4.8)	194 (5.4)
1c. Post‐operative characteristics			
ICU‐time			
≤1 day	22 136 (74.2)	19 608 (74.8)	2528 (70.1)
Missing	1013 (3.4)	856 (3.3)	157 (4.4)
Myocardial infarction			
Yes	793 (2.7)	681 (2.6)	112 (3.1)
Missing	1795 (6.0)	1554 (5.9)	241 (6.7)
Renal replacement therapy^e^			
Yes	1105 (3.7)	934 (3.6)	171 (4.7)
Missing	2000 (6.7)	1745 (6.7)	255 (7.1)
Reoperation			
Yes	1994 (6.7)	1,738 (6.6)	256 (7.1)
Missing	1055 (3.5)	893 (3.4)	162 (4.5)
Sternal wound infection			
Yes	388 (1.3)	320 (1.2)	68 (1.9)
Missing	1975 (6.6)	1718 (6.6)	257 (7.1)
Stroke			
Yes	600 (2.0)	523 (2.0)	77 (2.1)
Missing	1750 (5.9)	1511 (5.8)	239 (6.6)

Abbreviations: BMI, body mass index; CABG, coronary artery bypass grafting; ICU, intensive care unitIQR, interquartile range; n, number.

^a^ No opioid dispensing within six months before cardiac surgery.

^b^ Defined as serum creatinine > 200.

^c^ Includes pharmacologically treated diabetes mellitus type 1 and type 2.

^d^ Epidural catheters were used in only one of the four heart centers (Odense).

^e^ Includes hemodialysis, peritoneal dialysis, and continuous veno‐venous hemofiltration.

### 3.2 Chronic post‐operative opioid use

The overall proportion of chronic post‐operative opioid use was 10.6% (95% CI: 10.2‐10.9). The proportion of chronic post‐operative opioid use was 5.7% (95% CI: 5.5‐6.0) in pre‐operative opioid naïve patients, 68.3% (95% CI: 66.1‐70.4) in pre‐operative low dose opioid users, and 76.3% (95% CI: 74.0‐78.5) in pre‐operative high dose opioid users (Table 2).

**Table 2:** Post‐operative opioid use by pre‐operative opioid status, Western Denmark, 2003‐2016

Total, n	All patients, % (95% CI)	Opioid naïve^a^, % (95% CI)	Low dose opioid user^b^, % (95% CI)	High dose opioid user^c^, % (95% CI)
	29 815 (100)	26 206 (100)	27 827	28 302
Post‐operative quarter				
Q1	28.6 (28.1‐29.1)	24.2 (23.7‐24.7)	76.8 (74.8‐78.6)	82.6 (80.5‐84.5)
Q2	10.9 (10.6‐11.3)	5.9 (5.6‐6.2)	69.9 (67.8‐72.0)	77.3 (75.0‐79.4)
Q3	10.6 (10.2‐10.9)	5.6 (5.3‐5.9)	69.0 (66.8‐71.1)	76.2 (73.9‐78.4)
Q4	10.6 (10.2‐10.9)	5.7 (5.5‐6.0)	68.3 (66.1‐70.4)	76.3 (74.0‐78.5)

Abbreviations: CI, confidence interval; n, number; Q, quarter.

^a^ No opioid dispensing within six months before cardiac surgery.

^b^ Cumulative morphine equivalent opioid use within six months before cardiac surgery ≤ 500 mg.

^c^ Cumulative morphine equivalent opioid use within six months before cardiac surgery > 500 mg.

**Figure 2:**
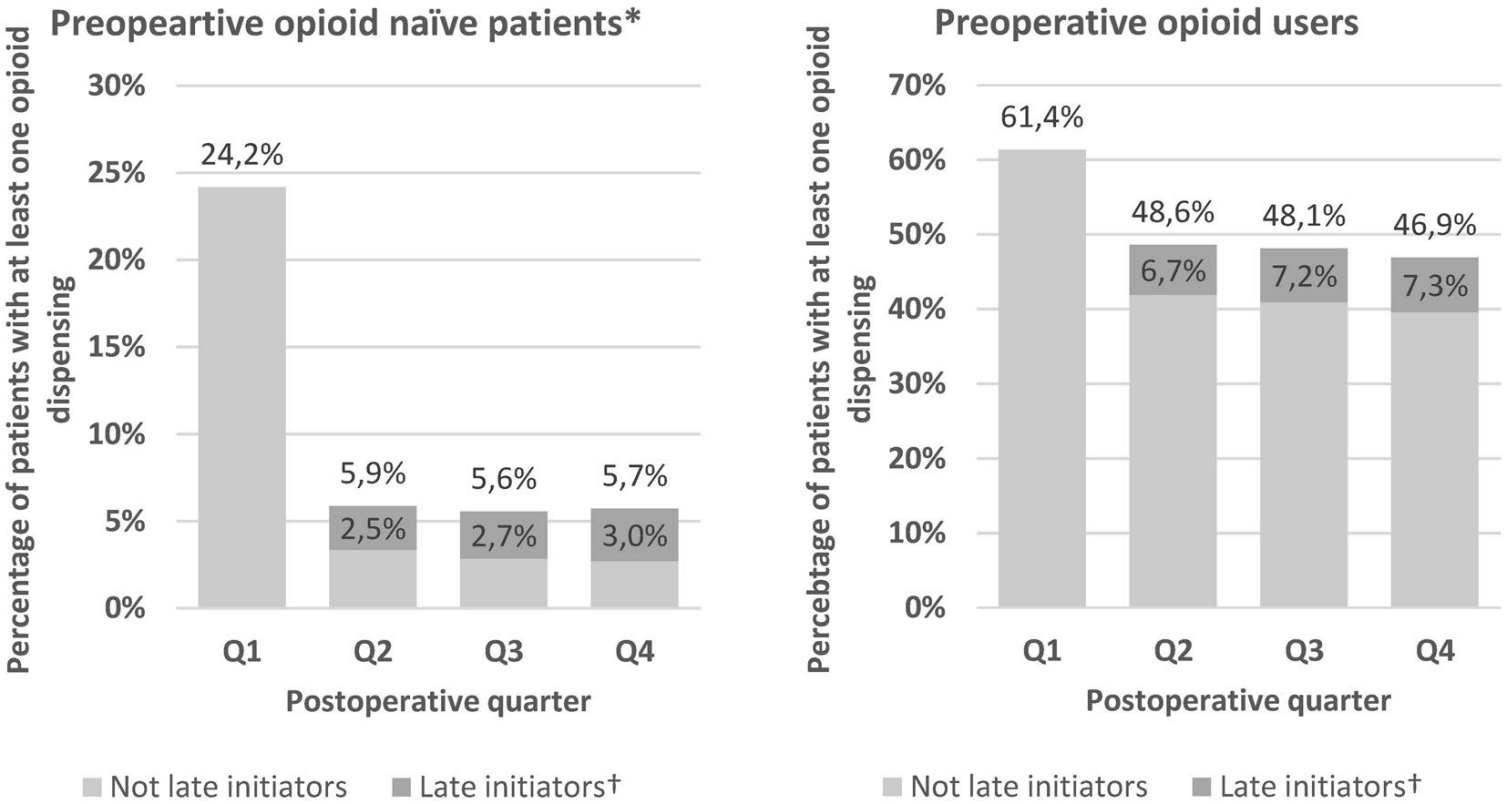
Percentage of patients with an opioid dispensing and the proportion attributed to late initiators in each postoperative quarter stratified by preoperative opioid status, western denmark, 2003–2016. Abbreviations: Q, quarter. *No opioid dispensing within six months before cardiac surgery. †Defined as first postoperative opioid dispensing ≥3 months after cardiac surgery

Among chronic post‐operative opioid users, late initiators (first opioid dispensing later than three months after surgery) accounted for 52.5% in pre‐operative opioid naïve patients and 15.6% in pre‐operative opioid users (Figure 2).

The overall proportions of chronic post‐operative opioid use was 10.9% (95% CI: 10.6‐11.3) in the second post‐operative quarter, 10.6% (95% CI: 10.2‐10.9) in the third post‐operative quarter, and 10.6% (95% CI: 10.2‐10.9) in the fourth post‐operative quarter. The proportion of opioid use remained steady from the second until the fourth post‐operative quarter for both pre‐operative opioid naïve patients, pre‐operative low dose opioid users, and pre‐operative high dose opioid users (Table 2).

Among chronic post‐operative opioid users, 57% had ≥ 5 opioid dispensings in the fourth post‐operative quarter; 12% had one dispensing (Figure ). The proportion of chronic post‐operative opioid users remained steady during the years studied (Figure 4). Opioid dispensing in the first post‐operative quarter decreased from 39% in 2003 to 29% in 2016. Tramadol accounted for approximately half of all opioid dispensings. Morphine, Oxycodone, and Codeine were also commonly dispensed (Table A3 in the appendix).

**Figure 3:**
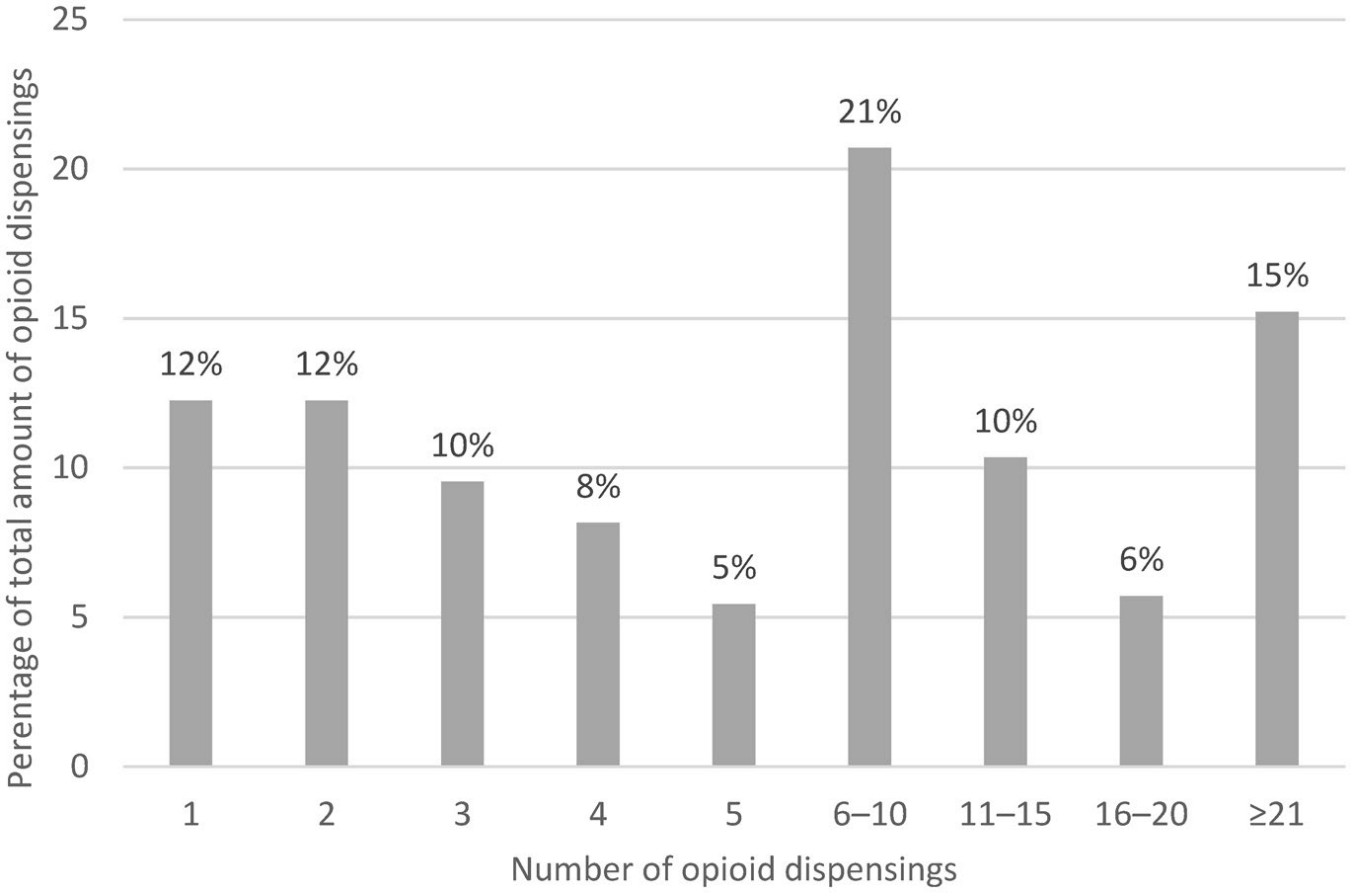
Number of opioid dispensings in the fourth postoperative quarter among chronic postoperative opioid users.

**Figure 4:**
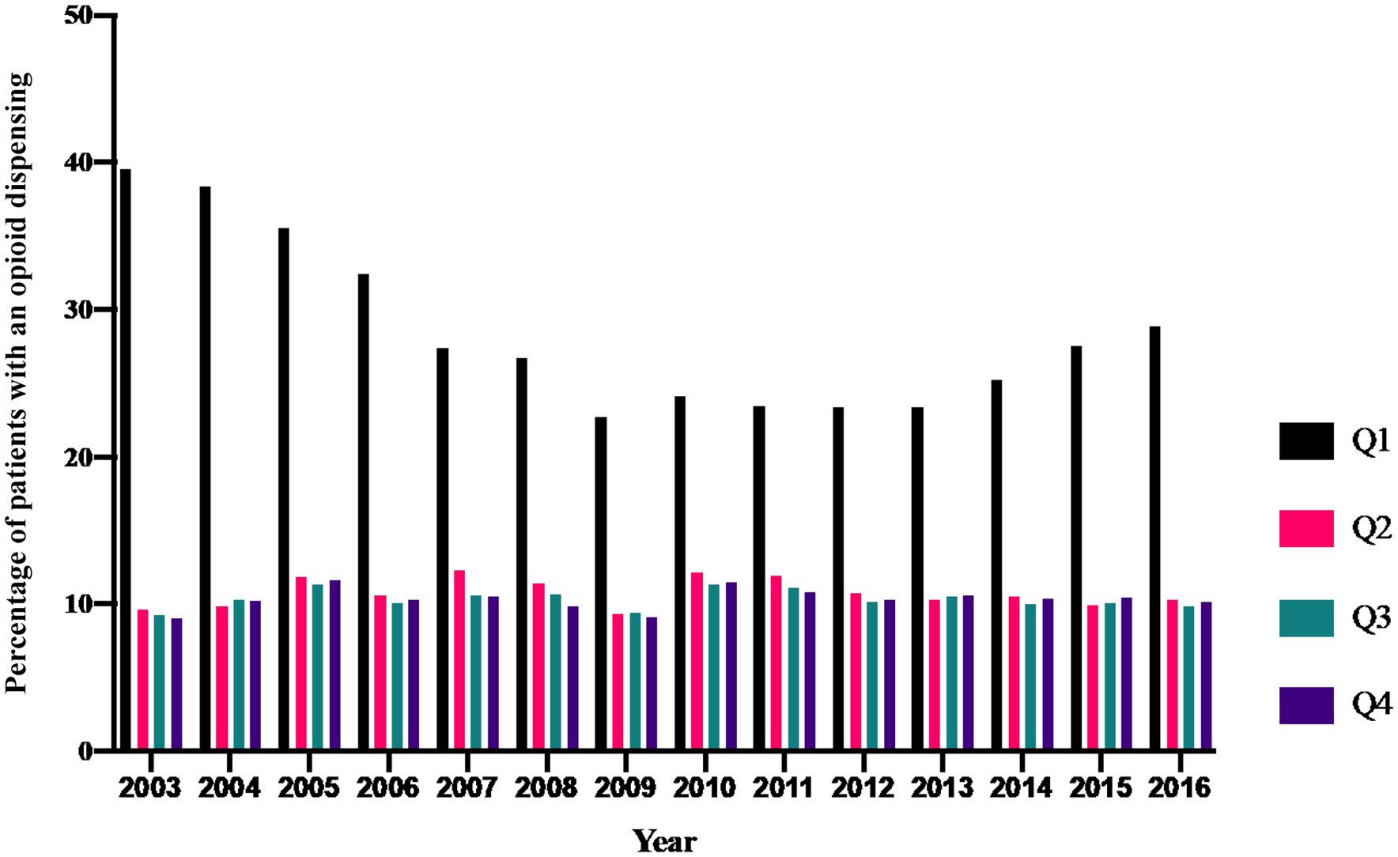
Percentage of patient with at least one opioid dispensing in each postopeartive quarter stratified by year Western Denmark, 2003‐2016. Abbreviations: Q, quarter

### 3.3 Risk factors for chronic post‐operative opioid use

**Figure 5:**
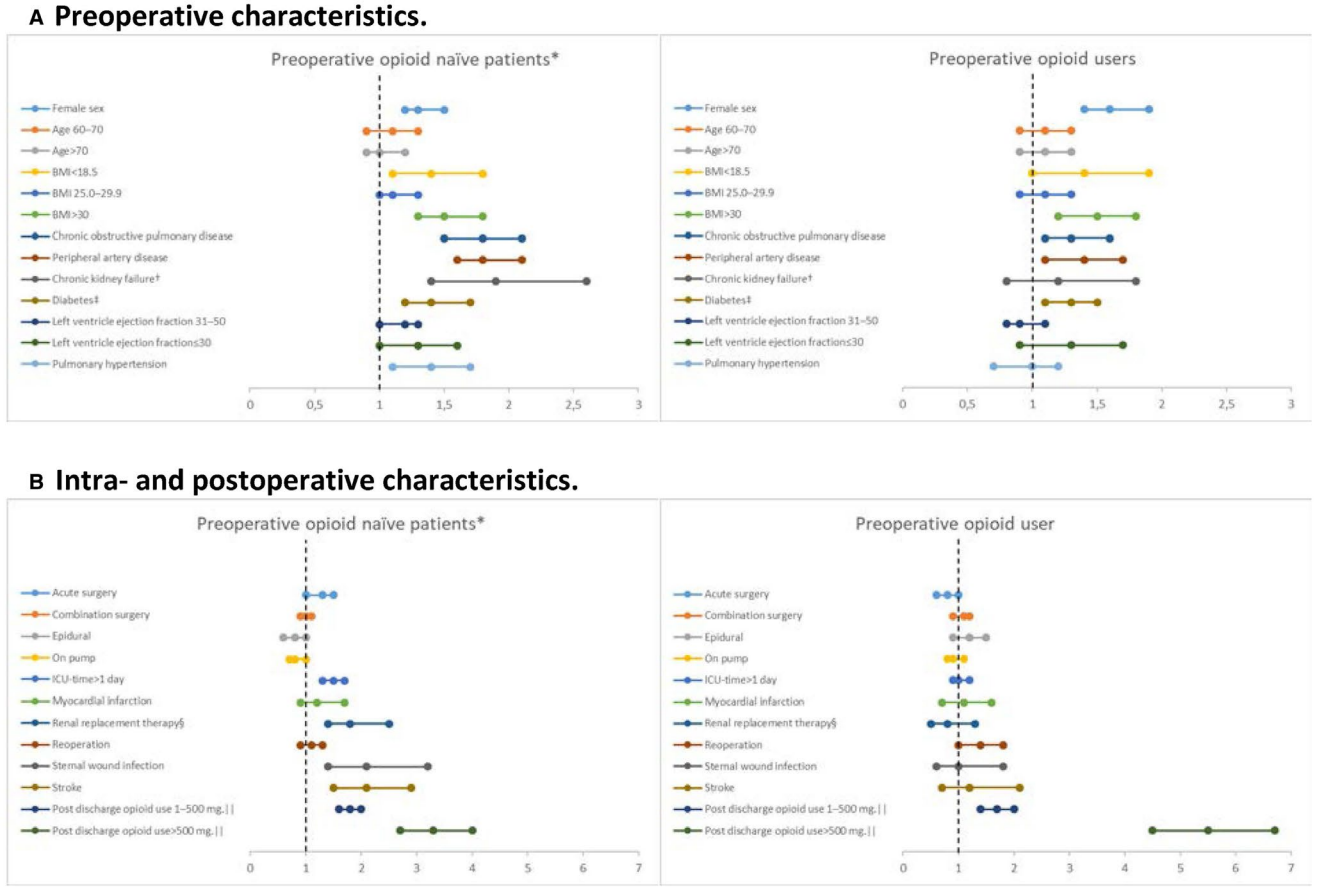
Odds ratios for pre‐, intra‐, and postopeartive risk factors for Abbreviations: CI, confidence interval; BMI, body mass index; ICU, intensive care unit at least one opioid dispensing in the forth postoperative quarter stratified by preopeartive opioid status. *No opioid dispensing within six months before cardiac surgery. †Defined as serum creatinine >200. ‡Includes pharmacologically treated diabetes mellitus type 1 and type 2. §Includes hemodialysis, peritoneal dialysis, and continuous veno‐venous hemofiltration. ||Cumulative morphine equivalent opioid use within one month after cardiac surgery

Pre‐operative opioid use was highly associated with chronic post‐operative opioid use (OR = 14.5, 95% CI: 13.3‐15.8). Cumulative post‐discharge OME use of more than 500 mg was highly associated with increased risk of chronic post‐operative opioid use in pre‐operative opioid naïve patients (OR = 3.3, 95% CI: 2.8‐4.0). Several pre‐, intra‐, and post‐operative factors were associated with increased risk of becoming a new chronic post‐operative opioid user including female gender, low and high BMI, pre‐operative comorbidities, acute surgery, prolonged ICU stay, and post‐operative complications such as sternal wound infection, need of renal replacement therapy, and stroke (Figure 5).

## 4 DISCUSSION

### 4.1 Opioid use distribution

In this retrospective study of opioid use after open cardiac surgery, we found chronic post‐operative opioid use, defined as at least one opioid dispensing in the fourth post‐operative quarter, in 5.7% (95% CI: 5.5‐6.0) of opioid naïve patients and 46.9% (95% CI: 45.2‐48.7) of pre‐operative opioid users. Opioid use remained steady as from the second post‐operative quarter.

To our knowledge, only two previous studies, both conducted in the United States, have investigated chronic post‐operative opioid use after cardiac surgery. One retrospective study followed 330 patients undergoing isolated coronary artery bypass grafting. Chronic post‐operative opioid use (defined as at least one opioid dispensing beyond 90 days after surgery) was 21.7% for pre‐operative opioid users and 3.2% and for opioid naïve participants (follow‐up time was one year).^27^ Comparison with the present study is hampered by the small study population, short follow‐up, and lack of data on actual opioid dispensing.^27^ Another retrospective study followed opioid naïve Medicare patients undergoing cardiothoracic surgery (coronary artery bypass grafting and/or valve repair/replacement) 6 months after surgery. Chronic post‐operative opioid use was seen in 12.8% of patients.^28^ Methodological differences rather than national differences in pain following cardiac surgery may explain this difference. First, these differences may be attributed to the timing and definition used for the primary outcome of chronic post‐operative opioid use. Second, the American population was limited to Medicare beneficiaries, with the inherent risk of selection bias. Finally, several studies have shown significant disparities between the quantity of opioids prescribed and actual consumption after surgery. A recent study described a median consumption of 27% of the opioid amount prescribed across 12 different surgical procedures.^29^ Classifying chronic post‐operative opioid use based on prescription dispensing according to the NPR may represent a more accurate measure of medication intake, even in the presence of some misclassification.^30^ The proportion of chronic post‐operative opioid users did not change during the study period. However, a decrease in opioid dispensing in the first post‐operative quarter was observed. Recent years’ increased awareness of the opioid epidemic may explain this trend, causing doctors to change their prescribing practice. Interestingly, this decrease did not continue into the following post‐operative quarters. The present data reflects opioid prescribing and chronic post‐operative opioid use prior to initiatives launched by the Danish Health Authority in 2017 (and going forward) with the purpose to reduce rates of chronic opioid use in Denmark. Most recent national data from 2017 on overall chronic opioid use indicates that chronic opioid use in Denmark has been reduced by 7%. Accordingly, rates of chronic post‐operative opioid use after cardiac surgery may be less today than described by present data.

The 5.7% incidence of new chronic post‐operative opioid users described in the present study is aligned with the reports published by Brummet et al describing that approximately 6% of patients undergoing other surgical conditions became new chronic opioid users. Our results support their conclusions that new chronic post‐operative opioid use is a common surgical complication that may be independent of the surgical burden.^16^ The stable opioid dispensing proportions from the second to the fourth post‐operative quarter along with the high number of opioid dispensing per patient both indicate an actual continuous opioid use among the individuals, and not just a single prescription for a potentially different cause.

### 4.2 Risk of opioid use

Pre‐operative opioid users had an almost 14‐fold increased risk of chronic post‐operative opioid use compared with opioid naïve patients. Among opioid naïve patients who were dispensed an opioid in the first post‐operative quarter, 76% were successful in becoming opioid‐free one year after surgery, in contrast with only 23% among opioid users. This is consistent with previous work that has identified pre‐operative opioid use as one of the strongest indicators of long‐term post‐operative use.^31^, ^32^ Known risk factors for new chronic post‐operative opioid use were further substantiated by the present study. These factors included female gender, BMI below 18.5, BMI above 30.0, and pre‐operative comorbidity. Contrary with existing literature, though still anticipated, was the association between intraoperative characteristics and immediate post‐operative complications and the onset of chronic post‐operative opioid use in pre‐operative naïve patients.

The use of intraoperative epidural catheter did show a trend toward a decreased risk of chronic post‐operative opioid use among pre‐operative opioid naïve patients. Assuming that epidural catheters provide optimal post‐operative pain treatment, this finding supports current evidence showing that adequate post‐operative treatment limits the risk of chronic post‐operative opioid use.^10^, ^11^ Epidural catheters were used only in one of the four heart centers. Therefore, we cannot exclude that non‐measured factors related to the operating hospital and the regional area other than use of epidural catheters explain the observed decrease risk of chronic post‐operative opioid use.

Data on prescribing behaviors during hospitalization were not available for the present study population. Hence, our study results do not elicit if special prescribing patterns in the immediate post‐operative period increased the risk of chronic post‐operative opioid use in pre‐operative naïve patients.

Opioid use within the first month from discharge was highly associated with chronic post‐operative opioid use in pre‐operative opioid naïve patients. Pre‐operative naïve patients receiving more than 500 mg OME had a more than threefold risk of becoming chronic post‐operative opioid users compared to patients with no post‐discharge dispensing. These findings are very much aligned with reports from cardiothoracic patients published by Brescia et al describing the adjusted rate of new chronic post‐operative users to be 19.6% among patients with intraoperative prescription size of more than 450 OME with a threshold effect between 300‐450 OME and > 450 OME.^28^ Our findings support their conclusion that post‐discharge opioid prescription is a modifiable predictor of new chronic post‐operative opioid use, and clinicians should certainly avoid prescribing more than 60 tablets of 5 mg Oxycodone for full sternotomy. However, more elaborated, patient‐tailored and evidence‐based prescribing guidelines are needed to respond to the risk of inducing new chronic post‐operative opioid users among adult cardiac surgery patients.

### 4.3 Clinical implications

Annually, more than 400 Danish patients become chronic post‐operative opioid users after cardiac surgery; noteworthy, many of these patients are new chronic opioid users. Preventive measures focused on patients who may be at higher risk for chronic post‐operative opioid use should be investigated and implemented. Possible modifying practices may include pre‐operative biopsychosocial programs to reduce opioid use in the post‐operative period,^33^ and implementation of non‐opioid pain treatment (eg regional nerve blocks in the intra‐ and immediate post‐operative period). Opioids may not even be needed at the time of discharge, and if they are needed, clinicians should complement careful discharge scripts.

### 4.4 Strengths and weaknesses

A major strength of the present study is its multi‐institutional study design covering an unselected patient population with free access to a universal healthcare system and with individual patient‐data linkage. Furthermore, prospective data collection in the WDHR and complete patient follow‐up minimized the risk of selection bias. The present study is based on prescription dispensings rather than on issued prescriptions, which provides an important advantage as a prescription dispensing is a better surrogate marker for actual drug intake than a written prescription.^24^ Another important feature of the NPR is its inclusion of drugs used by nursing home residents, which limits differential misclassification of exposure status due to frailty among elderly individuals.^7^

A limitation of this study is the lack of data on indication for opioid use. Thus, it is likely that some late initiators took opioids for pain conditions unrelated to the cardiac surgical procedure. Further limitations include unavailability of data on intended duration, and whether the drug was taken as recommended by the prescriber. Another limitation is the unavailability of data on inpatient opioid use and opioids dispensed in hospitals.

## 5 CONCLUSION

Chronic post‐operative opioid use was observed in 5.7% (95% CI: 5.5‐6.0) of pre‐operative opioid naïve patients, 68.3% (95% CI: 66.1‐70.4) in pre‐operative low dose opioid users, and 76.3% (95% CI: 74.0‐78.5) in pre‐operative high dose opioid users. Opioid use remained steady from the second post‐operative quarter. Pre‐operative opioid use and post‐discharge opioid dispensing was highly associated with increased risk of chronic post‐operative opioid use in pre‐operative opioid naïve patients, and may present as modifiable factors that need more clinical attention in order to avoid new chronic post‐operative opioid users among the cardiac surgery population.

## Supporting information

Supplementary Material

## Appendix

**Table A3:** Distribution of post‐operative opioid dispensings, Western Denmark, 2003‐2016

	Before cardiac surgery, n (%)	Post‐operative quarter, n (%)			
		Q1	Q2	Q3	Q4
Total	13 076 (100)	17 256 (100)	8461 (100)	8061 (100)	8261 (100)
Opioid agonists					
Morphine	1,268 (9.7)	2,229 (12.9)	1,073 (12.7)	1,054 (13.1)	1,067 (12.9)
Hydromorphone	<3 (0.0)	0 (0.0)	0 (0.0)	0 (0.0)	0 (0.0)
Nicomorphine	85 (0.7)	76 (0.4)	30 (0.4)	34 (0.4)	59 (0.7)
Oxycodone	1,699 (13.0)	3,649 (21.2)	1,491 (17.6)	1,395 (17.3)	1,439 (17.4)
Oxycodone and naloxone	8 (0.1)	5 (0.0)	4 (0.1)	3 (0.0)	4 (0.1)
Ketobemidone	<3 (0.0)	<3 (0.0)	0 (0.0)	0 (0.0)	0 (0.0)
Pethidine	168 (1.3)	84 (0.5)	102 (1.2)	82 (1.0)	48 (0.6)
Fentanyl	254 (1.9)	210 (1.2)	267 (3.2)	280 (3.5)	311 (3.8)
Dextropropoxyphene	129 (1.0)	48 (0.3)	52 (0.6)	43 (0.5)	44 (0.5)
Pentazocine	0 (0.0)	4 (0.0)	0 (0.0)	0 (0.0)	3 (0.0)
Ketobemidone and antispasmodic	732 (5.6)	1007 (5.8)	387 (4.6)	380 (4.7)	377 (4.6)
Codeine and paracetamol	1,076 (8.2)	1095 (6.4)	625 (7.4)	610 (7.6)	576 (7.0)
Codeine and acetylsalicylic acid	147 (1.1)	32 (0.2)	27 (0.3)	49 (0.61)	46 (0.6)
Dual‐action opioid agonists					
Tramadol	7,067 (54.1)	8,609 (49.9)	4,157 (49.1)	3,867 (48.0)	4,016 (48.6)
Tapentadol	19 (0.2)	9 (0.1)	8 (0.1)	15 (0.2)	13 (0.2)
Partial opioid agonists					
Buprenorphine	420 (3.2)	198 (1.2)	238 (2.8)	249 (3.1)	258 (3.1)

Abbreviations: n, number; Q, quarter.

**Table A4:** Pre‐operative risk factors of fourth quarter post‐operative opioid use stratified by pre‐operative opioid status, Western Denmark, 2003‐2016

Variable	Pre‐operative opioid naïve^a^, OR (95% CI)	Pre‐operative opioid user, OR (95% CI)
Gender		
Male	1 (ref.)	1 (ref.)
Female	1.3 (1.2‐1.5)	1.6 (1.4‐1.9)
Age		
<60	1	1
60‐70	1.1 (0.9‐1.3)	1.1 (0.9‐1.3)
>70	1.0 (0.9‐1.2)	1.1 (0.9‐1.3)
BMI		
18.5‐24.9	1	1
<18.5	1.4 (1.1‐1.8)	1.4 (1.0‐1.9)
25.0‐29.9	1.1 (1.0‐1.3)	1.1 (0.9‐1.3)
≥30.0	1.5 (1.3‐1.8)	1.5 (1.2‐1.8)
Chronic obstructive pulmonary disease		
No	1	1
Yes	1.8 (1.5‐2.1)	1.3 (1.1‐1.6)
Peripheral artery disease		
No	1	1
Yes	1.8 (1.6‐2.1)	1.4 (1.1‐1.7)
Chronic kidney failure^b^		
No	1	1
Yes	1.9 (1.4‐2.6)	1.2 (0.8‐1.8)
Diabetes^c^		
No	1	1
Yes	1.4 (1.2‐1.7)	1.3 (1.1‐1.5)
Left ventricle ejection fraction		
≥50	1	1
31‐50	1.2 (1.0‐1.3)	0.9 (0.8‐1.1)
≤30	1.3 (1.0‐1.6)	1.3 (0.9‐1.7)
Pulmonary hypertension		
No	1	1
Yes	1.4 (1.1‐1.7)	1.0 (0.7‐1.2)

Abbreviations: BMI, body mass indexCI, confidence interval; OR, odds ratio.

^a^ No opioid dispensing within six months before cardiac surgery.

^b^ Defined as serum creatinine > 200.

^c^ Includes pharmacologically treated diabetes mellitus type 1 and type 2.

**Table A5:** Intra‐ and post‐operative risk factors of fourth quarter post‐operative opioid use stratified by pre‐operative opioid status, Western Denmark, 2003‐2016

Variable	Preoperative opioid naïve^a^, OR (95% CI)	Preoperative opioid user, OR (95% CI)
Acute surgery		
No	1 (ref.)	1 (ref.)
Yes	1.3 (1.0‐1.5)	0.8 (0.6‐1.0)
Surgery type		
CABG, without valve surgery	1	1
Combinations and other	1.0 (0.9‐1.1)	1.1 (0.9‐1.2)
Epidural		
No	1	1
Yes	0.8 (0.6‐1.0)	1.2 (0.9‐1.5)
Extra corporeal circulation		
Off pump	1	1
On pump	0.8 (0.7‐1.0)	0.9 (0.8‐1.1)
**ICU‐time**		
≤1 day	1	1
>1 day	1.5 (1.3‐1.7)	1.0 (0.9‐1.2)
Myocardial infarction		
No	1	1
Yes	1.2 (0.9‐1.7)	1.1 (0.7‐1.6)
Renal replacement therapy^b^		
No	1	1
Yes	1.8 (1.4‐2.5)	0.8 (0.5‐1.3)
Reoperation		
No	1	1
Yes	1.1 (0.9‐1.3)	1.4 (1.0‐1.8)
Sternal wound infection		
No	1	1
Yes	2.1 (1.4‐3.2)	1.0 (0.6‐1.8)
Stroke		
No	1	1
Yes	2.1 (1.5‐2.9)	1.2 (0.7‐2.1)
Post‐discharge opioid use^c^		
0 mg.	1	1
1‐500 mg.	1.8 (1.6‐2.0)	1.7 (1.4‐2.0)
>500 mg.	3.3 (2.8‐4.0)	5.5 (4.5‐6.7)

Abbreviations: CI, confidence interval; OR, odds ratio.

^a^ No opioid dispensing within six months before cardiac surgery.

^b^ Includes hemodialysis, peritoneal dialysis, and continuous veno‐venous hemofiltration.

^c^ Cumulative morphine equivalent opioid use within one month after cardiac surgery.
