# Supplementary figures and images for: Chronic post‐operative opioid use after open cardiac surgery: A Danish population‐based cohort study
Source: Acta Anaesthesiol Scand. 2020 Sep 9;65(1):47–57. doi: 10.1111/aas.13688 (PMC7754369; doi:10.1111/aas.13688)

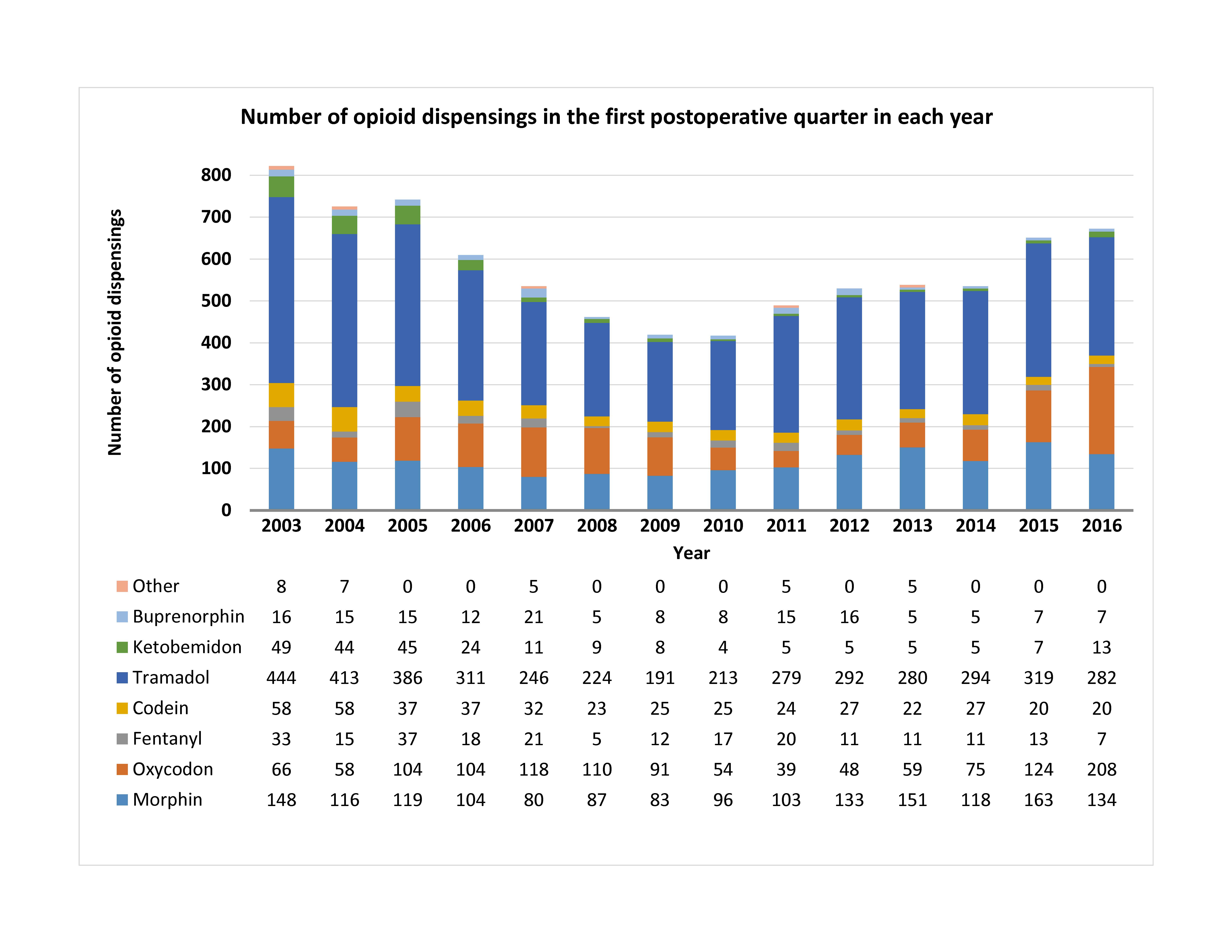

Supplement: Supplementary file 1 — Supplementary Material [file AAS-65-47-s001.jpg]
